# Integrating plasma proteomes with genome-wide association data for causal protein identification in multiple myeloma

**DOI:** 10.1186/s12916-023-03086-0

**Published:** 2023-09-29

**Authors:** Qiangsheng Wang, Qiqin Shi, Zhenqian Wang, Jiawen Lu, Jian Hou

**Affiliations:** 1 Department of Hematology, Ningbo Hangzhou Bay Hospital, Ningbo, 315000 Zhejiang China; 2 Department of Ophthalmology, Ningbo Hangzhou Bay Hospital, Ningbo, 315000 Zhejiang China; 3 School of Public Health (Shenzhen), Sun Yat-Sen University, Shenzhen, 518107 Guangdong China; 4 Department of Hematology, Renji Hospital, Shanghai Jiao Tong University School of Medicine, Shanghai, 200127 China

**Keywords:** Plasma proteome, Multiple myeloma, Drug target, Bayesian model, Mendelian randomization, Enrichment pathway analysis

## Abstract

**Background:**

Multiple myeloma (MM) is a severely debilitating and fatal B-cell neoplastic disease. The discovery of disease-associated proteins with causal genetic evidence offers a chance to uncover novel therapeutic targets.

**Methods:**

First, we comprehensively investigated the causal association between 2994 proteins and MM through two-sample mendelian randomization (MR) analysis using summary-level data from public genome-wide association studies of plasma proteome (*N* = 3301 healthy individuals) and MM (598 cases and 180,756 controls). Sensitivity analyses were performed for these identified causal proteins. Furthermore, we pursued the exploration of enriched biological pathways, prioritized the therapeutic proteins, and evaluated their druggability using the KEGG pathway analysis, MR-Bayesian model averaging analysis, and cross-reference with current databases, respectively.

**Results:**

We identified 13 proteins causally associated with MM risk (false discovery rate corrected *P* < 0.05). Six proteins were positively associated with the risk of MM, including nicotinamide phosphoribosyl transferase (NAMPT; OR [95% CI]: 1.35 [1.18, 1.55]), tyrosine kinase with immunoglobulin-like and EGF-like domains 1 (TIE1; 1.14 [1.06, 1.22]), neutrophil cytosol factor 2 (NCF2; 1.27 [1.12, 1.44]), carbonyl reductase 1, cAMP-specific 3',5'-cyclic phosphodiesterase 4D (PDE4D), platelet-activating factor acetylhydrolase IB subunit beta (PAFAH1B2). Seven proteins were inversely associated with MM, which referred to suppressor of cytokine signaling 3 (SOCS3; 0.90 [0.86, 0.94]), Fc-gamma receptor III-B (FCGR3B; 0.75 [0.65,0.86]), glypican-1 (GPC1; 0.69 [0.58,0.83]), follistatin-related protein 1, protein tyrosine phosphatase non-receptor type 4 (PTPN4), granzyme B, complement C1q subcomponent subunit C (C1QC). Three of the causal proteins, SOCS3, FCGR3B, and NCF2, were enriched in the osteoclast differentiation pathway in KEGG enrichment analyses while GPC1 (marginal inclusion probability (MIP):0.993; model averaged causal effects (MACE): − 0.349), NAMPT (MIP:0.433; MACE: − 0.113), and NCF2 (MIP:0.324; MACE:0.066) ranked among the top three MM-associated proteins according to MR-BMA analyses. Furthermore, therapeutics targeting four proteins are currently under evaluation, five are druggable and four are future breakthrough points.

**Conclusions:**

Our analysis revealed a set of 13 novel proteins, including six risk and seven protective proteins, causally linked to MM risk. The discovery of these MM-associated proteins opens up the possibility for identifying novel therapeutic targets, further advancing the integration of genome and proteome data for drug development.

**Supplementary Information:**

The online version contains supplementary material available at 10.1186/s12916-023-03086-0.

## Background

Multiple myeloma (MM) is a hematologic neoplasm caused by the malignant proliferation of clonal plasma cells. In 2019, more than 155,688 people were diagnosed with multiple myeloma worldwide, and approximately 100,000 deaths are attributed to MM per year [1]. Various pharmacological strategies have been developed against MM, including proteasome inhibitors, immunomodulatory agents, and alkylating agents, which have successfully increased patient survivorship [2]. However, most patients still experience relapse and resultant mortality due to drug resistance; therefore, the 5-year survival rate of MM patients in high-risk populations remains 50% or lower [3]. Hence, it is imperative to find novel therapeutic targets for the development of new anti-multiple myeloma agents.

Proteins are versatile biologically active compounds involved in the regulation of multiple cellular and physiological functions. A new generation of proteomics technologies has enabled the identification of ectopic protein expressions and further exploration of potential biomarkers and therapeutic targets for cancer [4]. For example, by employing a quantitative proteomics approach, Chen et al. recognized proteinase inhibitor 9 (SERPINB9) as a promising novel therapeutic target for bortezomib-resistant recurrent and relapsed MM [5]. More recently, a proteomic profiling analysis revealed that cyclin-dependent kinase 6 (CDK6) upregulation is a targetable resistance mechanism for lenalidomide, highlighting the expanding importance of proteomic research in MM [6]. However, most such studies were restricted to small sample sizes and/or limited protein species. And it is important to note that the causal relevance of associations from these nonrandomized observational studies remains largely unresolved due to their susceptibility to confounders or reverse causation.

Mendelian randomization (MR) is a popular approach for causal inferences by using genetic variants as instrumental variables (IVs) that mimic a lifetime randomized controlled trial [7]. It exploits the natural random allocation of genetic variants at conception, so results from MR are much less likely to be biased by reverse causation or residual confounding. With the development of genome-wide association studies (GWASs) on human plasma proteome, an optimization framework by integrating genomic and proteomic databases for biomarker discovery has emerged [8]. In particular, MR studies leveraging protein quantitative trait loci from variants have contributed to elucidating novel targets for breast cancer [9], lung cancer [10], and ovarian cancer [11], which suggests that the analytical method is empirically validated and reliable.

We therefore applied a proteome-wide MR analysis by combing the high-throughput proteomes with genetic data to assess the causal effects of the circulating proteins on the risk of MM. Furthermore, to explore the clinical utility of these proteins, we branch out the existing research and give out a three-step parallel approach: (i) revealing their roles in the etiology of MM; (ii) disentangling the prioritization of these proteins; and (iii) evaluating the druggability of potential target proteins.

## Methods

### Study design

**Fig. 1:**
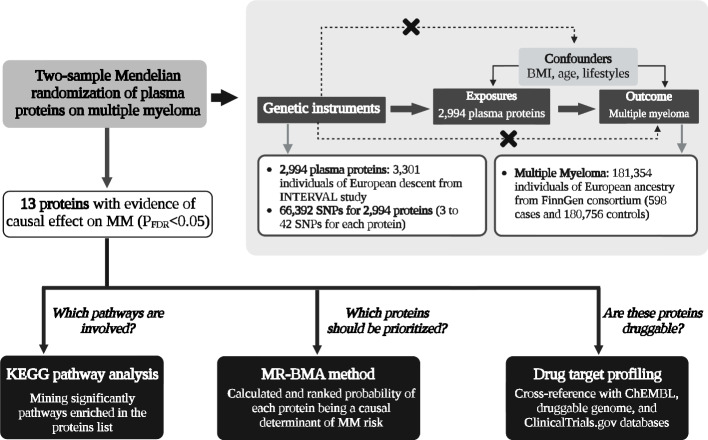
A flow chart of the study design and a schematic illustration of two-sample MR. BMI, body mass index; FDR, false discovery rate; GSMR, generalized summary-data-based Mendelian randomization; KEGG, Kyoto encyclopedia of genes and genome; MM, multiple myeloma; MR-BMA, MR-Bayesian model averaging; SNPs, single-nucleotide polymorphisms

The flow diagram summarizing the methodology of the study is depicted in Fig. 1 and the detailed information of the data source is displayed in Additional file 1: Table. S1. Firstly, we leveraged the two-sample MR method to explore the causal relationships between 2994 plasma proteome and the risk of MM using summary-level data from publicly GWASs of plasma proteome in INTERVAL study (*N* = 3301) [12] and MM in FinnGen consortium (598 cases and 180,756 controls) [13]. Three assumptions are required for the MR method: (1) the single nucleotide polymorphism (SNP) selected as the genetic instrument is robustly associated with the exposure (“relevance”); (2) the SNP is not associated with confounders (“exchangeability”); and (3) SNP affect the outcome only through the exposure (“exclusion restriction”) [14]. False discovery rate (FDR) correction was used to account for multiple testing. Second, we further explore the enriched pathways, priority of therapeutic targets for MM, and druggability in these causal proteins. To be specific, the Kyoto encyclopedia of genes and genome (KEGG) pathway analysis was performed to identify significantly enriched pathways in the causal proteins and MR-Bayesian model averaging (MR-BMA) was leveraged to rank causal proteins by their marginal score. To evaluate clinical development activity of candidate drugs targeting the candidate proteins, we comprehensively queried an updated list of druggable genes [15], the ChEMBL [16] database, and a clinical trials registry website.

### Plasma proteome GWAS data

The publicly available proteome GWAS dataset was derived from summary statistics of 2994 blood proteomes in 3301 healthy individuals of European ancestry from INTERVAL study [12]. For selection of individuals, Sun et al. randomly selected two non-overlapping sub-cohorts of 2731 and 831 from INTERVAL study and 3301 participants remained for analysis after genetic quality control. In the published GWAS study, relative concentrations of plasma proteins were measured by a multiplexed, aptamer-based approach (SOMAscan assay) and the reliability of protein measurements was validated using several subsequent experiments and replication of known associations with non-genetic factors. After quality control process, 3283 SOMA aptamers (SOMAmers) were left and mapped to 2994 unique plasma proteins in the final GWAS. In each sub-cohort, rank-inverse normalized protein residuals from linear regression of log-transformed protein levels on age, sex, duration between blood draw and processing, and the first three principal components were used as phenotypes and an additive genetic model was used to test genetic associations between 10.6 million imputed SNPs and levels of 2994 plasma proteins. Lastly, association results from the two sub-cohorts were combined using fixed-effects meta-analysis.

### MM GWAS data

GWAS data of MM used for primary analysis was extracted from summary statistics of 181,354 individuals of European ancestry from the FinnGen consortium (R5 release, https://finngen.gitbook.io/documentation/v/r5/) [13]. The FinnGen Study is a Finnish, nationwide GWAS meta-analysis of 9 biobanks, which was non-overlapped with the samples of plasma proteome GWAS. The biobanks have been linked with longitudinal digital health record data from national health registries. The GWAS of MM in the FinnGen Study included 598 cases and 180,756 individuals without cancers as controls. The MM was defined according to the international classification of diseases-10 (ICD-10) and ICD-9 in the record of hospital discharge registry and cause of death registry. The genetic associations between SNPs and MM were adjusted for age, sex, 10 principal components, and genotyping batch.

Another summary-level data on MM for sensitivity analysis was derived from a GWAS of 405,018 individuals of European ancestry from UK Biobank (552 cases and 404,466 controls) [17, 18]. MM was defined by electronic health records-derived ICD billing codes 204.4. Scalable and accurate implementation of generalized mixed model (SAIGE) with adjustment for genetic relatedness, sex, birth year, and the first 4 principal components was performed to test the genetic associations. There was also no overlap since samples of MM and plasma proteins were obtained from different cohorts.

### Selection of genetic instruments

To satisfy the assumptions of MR (Fig. 1), a set of criteria was drawn up. First, SNPs used as genetic instruments for each plasma protein were selected by a genome-wide significant level (*P* < 5 × 10^–8^). If the number of SNPs for the protein is less than 3, we adopted a suggestive genome-wide *P*-value threshold (*P* < 5 × 10^–6^) to identify enough SNPs (at least 3) in common between proteins and MM. Second, SNPs with linkage disequilibrium (LD) R^2^ > 0.001 within 10 Mb were excluded to ensure the independence of the SNPs. Third, SNPs with *F*-statistics < 10 were excluded to minimize instrument bias [19]. Fourth, we examined whether any of these SNPs were associated with confounders (age at recruitment, BMI, alcohol consumption, smoking, and physical activity) and outcomes at a *P*-value of Bonferroni level (0.05/number of SNPs) using the publicly available GWAS summary data. The associations of these SNPs with alcoholic drinks per week and smoking initiation were extracted from GWAS and sequencing consortium of alcohol and nicotine use (GSCAN) consortium [20]. Summary-level data of BMI and physical activity were derived from a meta-analysis of GWASs (~ 700,000 individuals) from genetic investigation of anthropometric traits (GIANT) consortium and UK Biobank and a GWAS (377,234 individuals) from UK Biobank, respectively [21, 22]. Given MM always occurs in old age, older populations have the competing risk of mortality and MM, which could create survival bias [23]. To alleviate the bias, we excluded the SNPs associated with survival which were proxied by age at recruitment, as described previously [24]. The associations of these SNPs with age at recruitment were derived from the MR-Base platform, including 361,194 individuals from UK Biobank [25]. Detailed information regarding these GWAS datasets is displayed in Additional file 1: Table. S1. Since no SNP were selected as genetic instruments for MM using the genome-wide significant threshold (*P* < 5 × 10^–8^), we also applied a less stringent threshold of 5 × 10^–6^ to obtain more SNPs for MM, as described before [24]. The remaining steps for the selection of genetic instruments for MM adhere to the aforementioned criteria.

### Statistical analysis

#### Power analysis

A priori statistical power was calculated by a web application (https://shiny.cnsgenomics.com/mRnd/) [26]. Assuming an alpha level of 5%, and a proportion of MM cases (1%), our analyses had a power of > 80% to detect a causal effect of OR = 1.30, when the protein explained by the SNPs (*R*^2^) is at least 5%.

#### Two-sample MR of plasma proteins on MM and sensitivity analyses

If a genetic instrument (SNP) was missing from the MM GWAS, the SNP was replaced by a proxy SNP in high LD *R*^2^ > 0.80 using LDlink (https://ldlink.nci.nih.gov/) [27]. Inverse-variance weighted (IVW) with random-effects method was leveraged to estimate causal effects of 2994 plasma proteins on the risk of MM. Random-effects IVW method was performed to take into account potential heterogeneity [28]. To account for multiple testing across 2994 plasma proteins analyses, an FDR-corrected *P*-value threshold of < 0.05 was established, which we used as a heuristic to define evidence for the association between protein and the risk of MM.

We conducted a series of sensitivity analyses to assess the robustness of our findings. First, we performed additional MR methods on our significant results from the IVW analyses, including MR-Egger, weighted median, and generalized summary-data-based MR (GSMR). MR-Egger was used to assess the presence of pleiotropic effects on MM, and the intercept distinct from zero provides evidence for pleiotropic effects [29]. An *I*^2^_GX_ was calculated to quantify the suitability of MR-Egger method and an *I*^2^_GX_ less than 0.9 indicates the presence of considerable risk of bias in the MR-Egger analysis [30]. Weighted median provided a robust result although half of genetic instruments are invalid or pleiotropic [31]. GSMR used the heterogeneity in dependent instruments (HEIDI)-outlier method to exclude outlier or heterogenous genetic instruments and accounts for LD among SNPs using the reference dataset for LD estimation [32]. In this study, the genotype data of Europeans from phase 3 of the 1000 Genomes project was used as the reference dataset. Additionally, Steiger filtering method was performed to identify whether there are reverse causal SNPs (i.e., those explaining more variance in the outcome than the exposure) [33]. The reverse causal SNPs violate the MR assumption of “exclusion restriction”. Second, we repeated the primary analysis using summary-level data of MM from UK Biobank and combined the primary results and results of UK Biobank using fixed-effects meta-analysis. Third, we leveraged two-sample MR method to assess whether clinically diagnosed MM showed evidence of causally impacting levels of significant proteins, rather than vice versa. Finally, where applicable, we performed two-sample MR using only SNPs in the *cis*-region of the gene encoding the protein (defined as within ± 1Mb window from the gene) to investigate whether the significant associations identified in our study were being driven by *cis*-regulator SNPs. Wald ratio method was used for one SNP and IVW method was conducted for more than one SNP.

#### KEGG pathway analysis

KEGG is an open and widely used database integrating information on genomes, biological pathways, diseases, and drugs. KEGG pathway analysis was performed to mine pathways enriched in the list of significant proteins. To account for multiple testing, the FDR corrected *P*-value on the pathway less than 0.05 was significant.

#### MR-BMA method

MR-BMA is a novel multivariable MR paradigm that ranks risk factors by the probability of that risk factor being a causal determinant of the outcome and selects causal risk factors for outcomes in a Bayesian framework from a high-dimensional set of related and potentially highly correlated candidate risk factors [34]. To overcome the high correlation of plasma proteins and “measured pleiotropy” caused by other significant proteins, MR-BMA was leveraged to identify the dominating proteins over the others and assess which candidate causal proteins should be prioritized for MM, as described before [34–36]. We integrated the significant protein-associated SNPs (according to the aforementioned criteria) as instrumental variables in MR-BMA because we wanted to disentangle their causal roles. In MR-BMA, the multivariable MR analysis using weighted regression was undertaken in multiple combinations of proteins, including all single protein, all pairs of proteins, and all triples. The goodness-of-fit of the regression model was assessed by posterior probability of that combination being the true causal proteins. Then, a score was assigned to each protein that was calculated by adding the posterior probabilities of models including that protein, which is called marginal inclusion probability (MIP) and represented the probability of that protein being a causal determinant of MM. Model-averaged causal effect (MACE) for each protein was calculated and represent conservative estimates of the direct causal effect of a protein on the MM averaged across these models. Empirical *P*-values for MIP of each protein are calculated using a permutation method, with adjustment for multiple testing via the false-discovery rate (FDR) correction. Subsequently, Cochran’s *Q*-statistic was used to identify outlying SNPs and Cook’s distance was used to identify influential SNPs in the visited MR-BMA models (posterior probability > 0.02). Finally, we re-run the MR-BMA analysis omitting influential SNPs and outliers as our primary analysis, as suggested previously [34, 35].

#### Evaluation of druggability

We evaluated the druggability of the candidate target proteins by querying a list of druggable genes [15], ChEMBL database (release 27) [16], and https://www.ClinicalTrials.gov website; 4479 druggable genes were shown in the list and assembled in three tiers, including targets of approved drugs and drugs in clinical development (tier 1), proteins closely related to drug targets or with associated drug-like compounds (tier 2), and extracellular proteins and members of key drug-target families (tier 3) [15]. ChEMBL is a large, open-access bioactivity database (https://www.ebi.ac.uk/chembl), and we retrieved the ChEMBL to obtain information on compound name, molecule type, action type, and clinical development activity of the targeted proteins. Additionally, we searched https://www.ClinicalTrials.gov to obtain the name and clinical phase of protein targeted drug, if applicable.

All analyses were two-sided and conducted using TwoSampleMR (version 0.5.6), GSMR (version 1.0.9) and clusterProfiler (version 3.14.3) packages in R software (version 3.6.3). R-code for MR-BMA was sourced from https://github.com/verena-zuber/demo_AMD.

## Results

### Causal effect of plasma proteins on MM

**Fig. 2:**
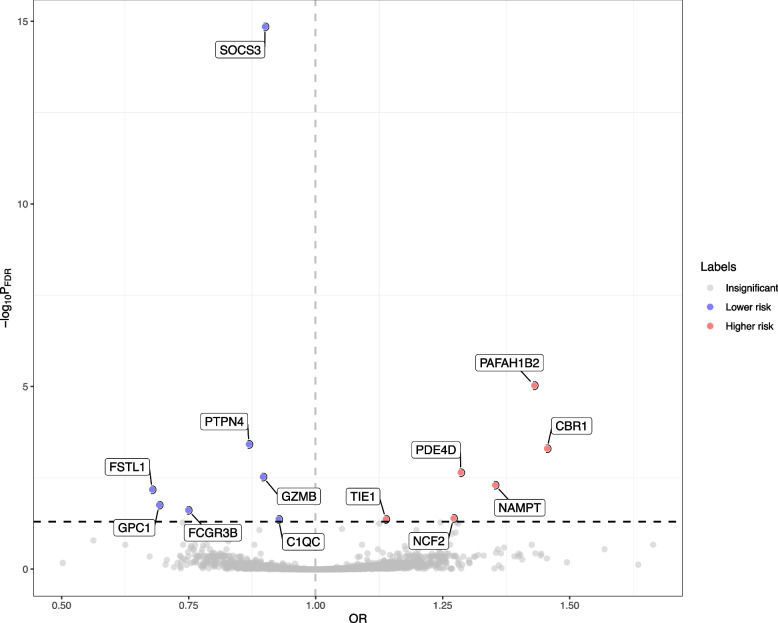
Volcano plot showing effects of human plasma proteins on the risk of multiple myeloma. Data are expressed as odds ratios (OR) estimated by the inverse variance-weighted (IVW) method. The red dots represent the plasma proteins was significant positively associated with the risk of multiple myeloma (*P*_FDR_ < 0.05). The blue dots represent the plasma proteins significant was inversely associated with the risk of multiple myeloma (*P*_FDR_ < 0.05). The black dashed line represents the association threshold of FDR corrected *P*-value < 0.05. C1QC, complement C1q subcomponent subunit C; CBR1, carbonyl reductase 1; FCGR3B, Fc-gamma receptor III-B; FSTL1, follistatin-related protein 1; FDR, false discovery rate; GPC1, glypican 1; GZMB, granzyme B; NAMPT, nicotinamide phosphoribosyl transferase; NCF2, neutrophil cytosol factor 2; PAFAH1B2, platelet-activating factor acetyl hydrolase IB subunit beta; PDE4D, cAMP-specific 3',5'-cyclic phosphodiesterase 4D; PTPN4, protein tyrosine phosphatase non-receptor type 4; SOCS3, suppressor of cytokine signaling 3; TIE1, Tyrosine kinase with immunoglobulin-like and EGF-like domains 1; SNP, single nucleotide polymorphism

**Fig. 3:**
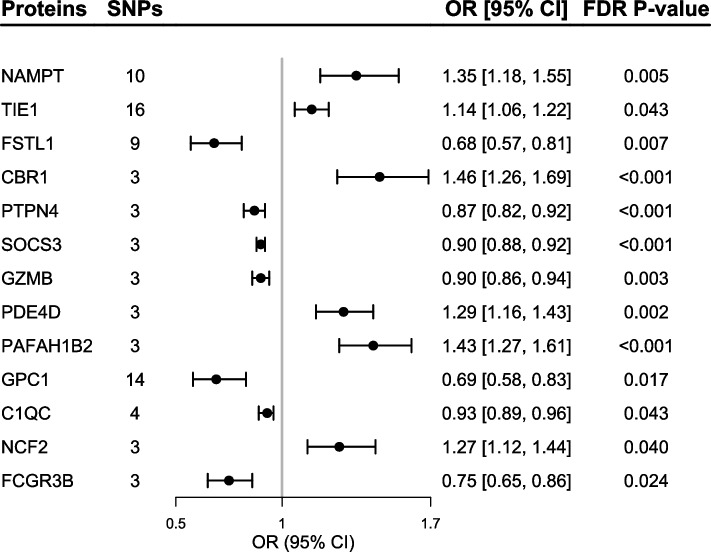
Mendelian randomization results of causal risk proteins on the risk of multiple myeloma. FDR *P*-value indicates the *P*-value was adjusted for multiple testing using false-discovery rate (FDR) method. C1QC, complement C1q subcomponent subunit C; CBR1, carbonyl reductase 1; FCGR3B, Fc-gamma receptor III-B; FSTL1, follistatin-related protein 1; FDR, false discovery rate; GPC1, glypican 1; GZMB, granzyme B; NAMPT, nicotinamide phosphoribosyl transferase; NCF2, neutrophil cytosol factor 2; PAFAH1B2, platelet-activating factor acetyl hydrolase IB subunit beta; PDE4D, cAMP-specific 3',5'-cyclic phosphodiesterase 4D; PTPN4, protein tyrosine phosphatase non-receptor type 4; SOCS3, suppressor of cytokine signaling 3; TIE1, Tyrosine kinase with immunoglobulin-like and EGF-like domains 1; SNP, single nucleotide polymorphism

Of the 2994 proteins being studied, the primary MR analysis showed causal relationships between 13 plasma proteins and the risk of MM (*P*_FDR_ < 0.05; Fig. 2). According to the criteria of genetic instrument selection, we selected 3 to 16 SNPs to genetically proxied 13 proteins (Additional file 1: Table. S2). Of note, the suppressor of cytokine signaling 3 (SOCS3) had the most significant MR result (OR [95% CI]: 0.90 [0.86, 0.94]; *P*_FDR_ < 0.001; Figs. 2 and 3). Except for SOCS3, primary MR analysis also showed genetically predicted higher levels of six proteins were associated with lower risk of MM, including protein tyrosine phosphatase non-receptor type 4 (PTPN4; 0.87 [0.82, 0.92]; *P*_FDR_ < 0.001), granzyme B (GZMB; 0.90 [0.86, 0.94]; *P*_FDR_ = 0.003), follistatin-related protein 1 (FSTL1; 0.68 [0.57, 0.81]; *P*_FDR_ = 0.007), glypican 1 (GPC1; 0.69 [0.58,0.83]; *P*_FDR_ = 0.017), Fc-gamma receptor III-B (FCGR3B; 0.75 [0.65,0.86]; *P*_FDR_ = 0.024), and complement C1q subcomponent subunit C (C1QC; 0.93 [0.89, 0.96]; *P*_FDR_ = 0.043; Fig. 3 and Additional file 1: Table. S3). Genetically predicted higher levels of six proteins were associated with a higher risk of MM, including nicotinamide phosphoribosyl transferase (NAMPT; 1.35 [1.18, 1.55]; *P*_FDR_ = 0.005), Tyrosine kinase with immunoglobulin-like and EGF-like domains 1 (TIE1; 1.14 [1.06, 1.22]; *P*_FDR_ = 0.043), carbonyl reductase 1 (CBR1; 1.46 [1.26, 1.69]; *P*_FDR_ < 0.001), cAMP-specific 3',5'-cyclic phosphodiesterase 4D (PDE4D; 1.29 [1.16, 1.43]; *P*_FDR_ = 0.002), platelet-activating factor acetylhydrolase IB subunit beta (PAFAH1B2; 1.43 [1.27, 1.61]; *P*_FDR_ < 0.001), and neutrophil cytosol factor 2 (NCF2; 1.27 [1.12, 1.44]; *P*_FDR_ = 0.040; Fig. 3 and Additional file 1: Table. S3).

### Pathway analysis

A KEGG pathway analysis was conducted. Three of 13 causal proteins were significantly enriched in the pathway of osteoclast differentiation (hsa04380; *P*_FDR_ = 0.020), including SOCS3, FCGR3B, and NCF2.

### MR-BMA analysis to rank causal proteins on MM

We applied MR-BMA in 13 causal proteins to disentangle their causal roles. We integrated the genetic instruments of 13 proteins and pruned the SNPs at an LD threshold of *r*^2^ < 0.001, leaving 75 SNPs for MR-BMA analysis (Additional file 1: Table. S4). After initially performing the MR-BMA method, ten models with posterior probability larger than 0.02 were selected to calculate Cochran’s Q statistic and Cook’s distance for each SNP. An influential SNP (rs10919543) was identified (Additional file 1: Table. S5) and no outliers were identified (Additional file 1: Table. S6). After removing the influential SNP, MR-BMA analysis was repeated and the top 10 models ranked according to their posterior probability were provided in Additional file 1: Table. S7. GPC1 (MIP:0.993; MACE: − 0.349; *P*_FDR_ = 0.013), NAMPT (MIP: 0.433; MACE: − 0.113; *P*_FDR_ = 0.022), and NCF2 (MIP: 0.324; MACE: 0.066; *P*_FDR_ = 0.022) are ranked in top-three among the 13 proteins (Table 1). It is noted that MR-BMA is designed to rank the causal importance of the 13 proteins. As such, when the *P*_FDR_ is greater than 0.05, it does not imply the absence of causality between those proteins and MM risk. Instead, it indicates that the rank of the subsequent 10 proteins remains uncertain.

**Table 1:** Top 10 causal proteins ranked by the marginal inclusion probability in the MR-BMA after model diagnostics

**Proteins**	**Marginal inclusion probability**	**Model-averaged causal effect**	**Empirical *P*-value**	**FDR *P*-value**
GPC1	0.993	− 0.349	0.001	0.013
NAMPT	0.433	0.113	0.005	0.022
NCF2	0.324	0.066	0.005	0.022
PDE4D	0.309	0.079	0.063	0.174
FSTL1	0.165	− 0.048	0.122	0.243
CBR1	0.157	0.049	0.131	0.243
TIE1	0.136	0.016	0.067	0.174
PTPN4	0.041	− 0.008	0.988	1.000
PAFAH1B2	0.031	− 0.005	0.997	1.000
SOCS3	0.028	− 0.003	0.948	1.000

Marginal inclusion probability for the protein, representing the probability of that protein being a causal determinant of MM risk. Model-averaged causal effect represents the average causal effect across models including that protein. Empirical *P*-values are computed using 1000 permutations and adjusted for multiple testing using false-discovery rate (FDR) procedure

*CBR1* carbonyl reductase 1, *FSTL1* follistatin-related protein 1, *FDR* false discovery rate, *GPC1* glypican 1, *NAMPT* nicotinamide phosphoribosyl transferase, *NCF2* neutrophil cytosol factor 2, *PAFAH1B2* platelet-activating factor acetyl hydrolase IB subunit beta, *PDE4D* cAMP-specific 3',5'-cyclic phosphodiesterase 4D, *PTPN4* protein tyrosine phosphatase non-receptor type 4, *SOCS3* suppressor of cytokine signaling 3, *TIE1* tyrosine kinase with immunoglobulin-like and EGF-like domains 1, *SNP* single nucleotide polymorphism

### Druggability and clinical-phase drug for candidate protein targets

We comprehensively searched a list of druggable genes [15], the ChEMBL (release 27) database [16], and the clinical trial registry website to evaluate the druggability and drug development of the 13 candidate proteins. We categorized candidate targets into three statuses, including approved, in development (in the phase of clinical trials), and druggable (listed as druggable targets). Notably, NAMPT-targeted drug DAPORINAD is entering phase I/II trials for cutaneous T-cell Lymphoma, melanoma, and B-cell chronic lymphocytic leukemia (Table 2). Additionally, NAMPT-target drugs TEGLARINAD, ATG-019, and KPT-9274 were all in the phase I trial for non-Hodgkin’s lymphoma or acute myeloid leukemia (Table 2). FCGR3B-targeted drug IMGATUZUMAB was currently being evaluated in clinical trials for colorectal neoplasms, head and neck neoplasms, and non-small-cell lung and PED4D inhibitor was currently being evaluated in clinical trials for Alzheimer’s disease, fragile X syndrome, and depression (Table 2). Although no ongoing trials for FSTL1, CBR1, GZMB, GPC1, and C1QC, they are potential druggable targets (Table 2). NCF2, PTPN4, SOCS3, and PAFAH1B2 are not currently listed as potential drug targeted according to the druggable gene list, ChEMBL database, and clinical trial registry website. However, enforced expression of SOCS3 is proposed as a potential treatment for triple-negative breast cancer and hepatocellular carcinoma [37, 38].

**Table 2:** Summary of druggability and clinical development activity for multiple myeloma associated with causal associations on Mendelian randomization analysis

**Target**	**Status**	**Compound name**	**Molecule type**	**Action type**	**Clinical development activities**
NAMPT	In development	DAPORINAD^b^ (CHEMBL566757)	Small molecule	Inhibitor	Phase I/II trials: cutaneous T-cell Lymphoma, melanoma, B-cell chronic lymphocytic leukemia
	In development	TEGLARINAD^b^ (CHEMBL1181731)	Small molecule	Inhibitor	Phase I trial: lymphoma
	In development	ATG-019^c^	Small molecule	Inhibitor	Phase I trial: non-Hodgkin's lymphoma (NCT04281420)
	In development	KPT-9274^c^	Small molecule	Inhibitor	Phase I trial: non-Hodgkin's lymphoma, acute myeloid leukemia (NCT02702492)
TIE1	In development	CP-459632^b^ (CHEMBL3545300)	Small molecule	Inhibitor	-
FSTL1	Druggable^a^	-	Antibody^a^	-	-
CBR1	Druggable^a^	-	Small molecule^a^	-	-
GZMB	Druggable^a^	-	Small molecule^a^	-	-
PDE4D	In development	ZATOLMILAST^b^ (CHEMBL4541964)	Small molecule	Inhibitor	Phase II trials: Alzheimer’s disease, fragile X syndrome, depression
GPC1	Druggable^a^	-	Antibody^a^	-	-
C1QC	Druggable^a^	-	Antibody^a^	-	-
FCGR3B	In development	IMGATUZUMAB^b^ (CHEMBL2109389)	Antibody	Cross-linking agent	Phase I/II trials: colorectal neoplasms, head and neck neoplasms, non-small-cell lung
NCF2	Not currently listed as druggable	-	-	-	-
PTPN4	Not currently listed as druggable	-	-	-	-
SOCS3	Not currently listed as druggable	-	-	-	-
PAFAH1B2	Not currently listed as druggable	-	-	-	-

*C1QC* complement C1q subcomponent subunit C, *CBR1* carbonyl reductase 1, *FCGR3B* Fc-gamma receptor III-B, *FSTL1* follistatin-related protein 1, *FDR* false discovery rate, *GPC1* glypican 1, *GZMB* granzyme B, *NAMPT* nicotinamide phosphoribosyl transferase, *NCF2* neutrophil cytosol factor 2, *PAFAH1B2* platelet-activating factor acetyl hydrolase IB subunit beta, *PDE4D* cAMP-specific 3',5'-cyclic phosphodiesterase 4D, *PTPN4* protein tyrosine phosphatase non-receptor type 4, *SOCS3* suppressor of cytokine signaling 3, *TIE1* tyrosine kinase with immunoglobulin-like and EGF-like domains 1, *SNP* single nucleotide polymorphism

^a^Data from druggable list [15]

^b^Data from ChEMBL release 27 [16] (compound ID in brackets)

^c^Data from https://www.ClinicalTrials.gov (clinical trial ID in brackets)

### Sensitivity analysis

To further increase robustness of the findings, we performed a set of sensitivity analyses. First, GSMR method showed consistent results with primary MR analysis (Additional file 1: Table. S3). Although MR-Egger and weighted methods showed no evidence of the associations between certain proteins and MM, the directions of the effects were consistent with the primary results, indicating the primary results were not substantially altered (Additional file 1: Table. S3). Moreover, *I*^2^_GX_ for MR-Egger estimates of NAMPT, FSTL1, CBR1, PTPN4, PAFAH1B2, and GPC1 is less than 90%, indicating MR-Egger estimates for the effect of six proteins on MM to be biased. MR-Egger intercept test indicated no evidence of horizontal pleiotropy (all *P*-values for intercept > 0.05; Additional file 1: Table. S3). Furthermore, GSMR method detected no HEIDI-outliers, indicating no evidence of pleiotropy (Additional file 1: Table. S3). In addition, all IVs showed the correct causal direction in the Steiger filtering analysis (i.e., IVs explain more variance in the exposure than the outcome; Additional file 1: Table. S2). Second, MR-IVW analysis of 13 proteins on MM was performed using the GWAS of MM from UK Biobank. With the exception of FSTL1, the effect directions of the other 12 proteins on MM in the UK Biobank were consistent with the primary results obtained from FinnGen consortium (Additional file 1: Table S8). Similarly, fixed-effects meta-analysis showed combined effects of 12 proteins on MM was significant and consistent with the primary results (*P* < 0.05), except for FSTL1 (*P* = 0.579; Additional file 1: Table. S8). Third, we investigated the causal relationships between MM and 13 proteins. According to the criteria of genetic instrument selection, we selected 8 SNPs for MM (Additional file 1: Table. S9). IVW showed no evidence of relationships between genetic predisposition to MM and 13 proteins, and MR-Egger, weighted median, and GSMR also showed concordant results (Additional file 1: Table. S10). Finally, we performed MR analyses using only *cis*-SNPs from all significant proteins. Out of 13 proteins, only six proteins have at least one *cis*-SNP and could therefore be analyzed. The results showed CBR1, GZMB, GPC1, C1QC, and FCGR3B were causally associated with the risk of MM (Additional file 1: Table. S11). All other significant associations might be driven by *trans*-SNPs.

## Discussion

In the present study, a pipeline composed of analytical techniques was utilized to analyze 2994 circulating proteins in relation to MM. The primary two-sample MR analysis revealed that 13 proteins were causally correlated with MM risk, with 6 showing positive associations (NAMPT, TIE1, CBR1, PDE4D, PAFAH1B2, and NCF2) and 7 showing inverse associations (FSTL1, PTPN4, SOCS3, GZMB, GPC1, C1QC, and FCGR3B). These included association with MM has been implicated elsewhere, such as NAMPT [39], PDE4D [40], PAFAH1B2 [41], SOCS3 [42], and GZMB [43]. The next step was the KEGG enrichment analysis, which showed that three of the causally associated proteins, SOCS3, FCGR3B, and NCF2, were enriched in the osteoclast differentiation pathway. Subsequently, MR-BMA analysis indicated that NAMPT, GPC1, and NCF2 ranked among the top three MM-associated proteins. At last, a list of 4 in-development protein-targeted drugs and 5 druggable proteins supported the incorporation of genomics and proteomics in the drug development programs again. Taken together, these findings exemplify the utility of genetic analysis in identifying both known and novel loci and pathways with causal implications for MM.

The etiology of MM is intricate, encompassing the dysfunction of multiple genes and signaling pathways as well as the abnormal regulation of cellular processes. Multiple lines of evidence have underscored a bidirectional prosurvival regulatory loop exists between osteoclasts (OCs) and MM cells in the bone marrow microenvironment [44]. On one hand, OCs exert immunomodulatory effects via upregulating various inhibitory checkpoint molecules and immune-suppressive cytokines, contributing to the immunosuppressive microenvironment in MM [45]. On the other hand, MM cells drive OCs formation and activation while hampering OCs generation and function. This cascade leads to bone resorption, impedes osteoblast activity, ultimately resulting in bone destruction and osteoporosis [46]. Therefore, numerous studies are exploring signaling molecules in OCs differentiation as potential therapeutic avenues for MM treatment, with denosumab serving as an illustrative example due to its effective capability in delaying and mitigating bone-related events [47, 48]. Our results consistently point to an enrichment of proteins associated with OCs differentiation, specifically SOCS3, FCGR3B, and NCF2, in MM. SOCS3, as a member of the suppressor family of cytokine signaling, acts to inhibit the activation of the Janus kinase (JAK)-signal transducer and activator of transcription (STAT) pathway. By negatively regulating the central JAK-STAT pathway, SOCS3 can further orchestrate bone cell growth, differentiation, and maintenance [49, 50]. Similarly, FCGR3B is the only inhibitory member of the FcγR immunomodulator family. Recent evidence suggests that cross-regulation of immunoreceptor tyrosine-based activation motif and Fc-γ receptors could promote the suppression of spleen tyrosine kinase activation, thus leading to the inhibition of osteoclast differentiation [51]. Finally, the gene encoding NCF2 also encodes the niacinamide adenine dinucleotide phosphate oxidase complex, thereby indirectly inducing osteoclast differentiation [52]. Collectively, our results lend support to the causal roles of these proteins and corroborate the significance of osteoclast differentiation in the etiology of MM.

Furthermore, given the interrelated nature of protein characteristics, a MR-BMA analysis was conducted to identify the priority causal proteins. It should be noted that the primary objective of this methodology is to detect causal risk factors among a high-dimensional set of candidates rather than to unbiasedly estimate the magnitude of their causative effects [34]. So, our results highlight the need for prioritization of NAMPT, GPC1, and NCF2 as they may be more proximal to the occurrence of MM. In detail, NAMPT serves as a rate-limiting enzyme in the salvage pathway of nicotinic acid dinucleotide synthesis [53]. In line with our findings, a recent study found that OT-82 exhibits a potent effect on MM, which can be attributed to its ability to induce cell death through the inhibition of NAMPT [54]. In addition, NAMPT is currently in clinical trials for hematological malignancies such as lymphoma, non-Hodgkin’s lymphoma, and acute myeloid leukemia. For NCF2, early studies specified that NCF2 is overexpressed in gastric cancer and promotes the progression of gastric cancer by activating the NF-kB signaling pathway [55]. Recently, there is emerging evidence that high expression of NCF2 is associated with poor prognosis in patients suffering from acute myeloid leukemia [56]. So, with its involvement in osteoclast differentiation as mentioned above, NCF2 is poised to play a vital role in the underlying mechanisms of MM. For GPC1, the available literature provides conflicting information on the role of GPC1. The previous perception of GPC1 as a biomarker for prostate cancer has been challenged by recent findings that reveal its complex, paradoxical role in the regulation of prostate cancer cell proliferation and migration [57]. Thus, despite being considered as a potential target for cancer therapy in some solid tumors, the actual application of targeting GPC1 has not been realized. Furthermore, new evidence suggests that GPC1 expression in bone marrow-derived stromal cells exerts inhibitory effects on cancer cells, making GPC1 a promising target for the development of anti-cancer therapies targeting fibroblast cells [58]. Nonetheless, the involvement of these proteins in MM may be substantial and merits further research attention.

In addition to the above proteins, the significance of other proteins in MM should not be disregarded. According to our druggable list, there are ongoing efforts to develop drugs that specifically target TIE1 and PDE4D. TIE1 is a tyrosine kinase receptor expressed by endothelial and hematopoietic cells and is functionally involved in major vascular diseases like atherosclerosis and tumor angiogenesis [59]. Despite the lack of precise information on clinical applications, numerous studies view TIE1 inhibitors as a potential therapeutic approach for antiangiogenic treatment [59, 60]. PDE4D, a primary cAMP-hydrolyzing enzyme in cells, is also a promising drug target. Studies have demonstrated therapeutic benefits of PDE4D inhibitors in the treatment of Alzheimer’s disease, Huntington’s disease, schizophrenia, and depression [61]. However, recent studies have indicated that targeting PDE4D can be used for the treatment of ER positive breast cancer [62], prostate cancer [63], or hepatocellular carcinoma [64]. Future research could explore the potential of PDE4D inhibitors for the treatment of MM. Furthermore, our findings indicate that CBR1, FSTL1, C1QC, and GZMB possess potential for pharmacological and clinical utilization and may be targeted through the use of small molecules or antibodies. The association of CBR1 with cancer has been extensively studied, especially with the recent discovery of its high expression in Philadelphia-like B-line acute lymphoblastic leukemia [65]. Likewise, it has been demonstrated that FSTL1 can suppress the proliferation of nicotine-induced lung cancer cells [66], and C1QC has proven valuable for the diagnosis of skin cutaneous melanoma with improved overall survival [67]. Furthermore, GZMB, as a crucial component in natural killer cells, has made a significant contribution to the treatment of MM [43]. Finally, the limited research on PAFAH1B2 or PTPN4 hinders the acquisition of extensive knowledge on their effects on MM. Nonetheless, PAFAH1B2 expression has been reported as a prognostic marker for MM in validation analysis [41] while PTPN4 has been found to serve as an upstream therapeutic target in the treatment of prostate cancer [68], indicating the potential for the two proteins as future research entry points.

Our study has several advantages. We innovatively explored a prospective way to intervene with circulating proteins to lower MM risk by studying the enriched pathway, the priority of therapeutic targets, and druggability of the potential causal proteins. Benefiting from the large-scale and non-overlapped GWASs data of proteome and MM, we could incorporate more functional proteins into our study and obtain more powerful MR estimates. Further, during the actual execution, a state-of-art method MR-BMA was conducted to probe the prioritized proteins and existing databases were comprehensively searched to depict the druggability profile of target proteins. We gained an advantage by applying a suggestive genome-wide *P*-value threshold (5 × 10^–6^) during the selection of genetic instruments, enabling the inclusion of a broader range of analyzable candidate proteins compared to the conventional standard of 5 × 10^–8^ [24, 69]. Lastly, the bidirectional MR analysis adds strength to the robustness of our findings by indicating that reverse causation is unlikely to have influenced the observed associations.

Several limitations need to be considered when interpreting our findings. First, our study was conducted using overall MM without specifying disease subtypes characterized by the immunoglobulin. Given the etiologic and prognostic heterogeneity within each subtype symptom, it is desirable to identify subtype-specific causal proteins. However, such analyses are currently constrained by the limited availability of genetic data underlying each MM subtype. Second, the causal estimation of several proteins on MM was not fully confirmed by MR-Egger method and weighted median method. However, it is important to highlight that the direction of estimates mostly aligns with the primary MR method (i.e., IVW method), and additional assessments such as Steiger filtering, GSMR HEIDI-outlier test, MR-Egger intercept test, and Cochran’s Q test did not reveal any evidence of invalid SNPs. Consequently, despite the lack of corroboration, these collective results serve to reinforce the robustness of our findings. These results still enhance the robustness of our findings. Third, MR is not perfectly analogous to a randomized controlled trial (RCT). Therefore, effects of potential causal proteins on MM derived from MR analyses may differ in magnitude from those anticipated in an RCT, and should be interpreted as life-course effects. But that does not contradict our intention to employ MR as an expedited approach to complement clinical trials and enhance their reliability. Fourth, the non-linear effects of some proteins on MM risk cannot be excluded. It is intriguing to consider the possibility that a protein may impact MM risk at extremely low or high levels, but detecting such effects in practical clinical settings can be challenging. Fifth, it is possible that the null effects of certain proteins on MM we observed may have been a consequence of inadequate statistical power due to the power of 80% to detect an OR of at least 1.30, considering *R*^2^ = 5%, while the proportion of variance explained by SNPs for certain proteins is less than 5% [12]. In addition, the only protein with a difference between the FinnGen Consortium and the UK Biobank dataset, FSTL1, should also be further investigated in a larger MM cohort. Thus, replication in larger studies of MM would be worthwhile. Finally, our analysis was confined to European ethnicity, and race issues frequently lead to the underuse of treatment and unintended interruptions in MM treatment [70]; thus, we need to be careful in generalizability of our findings to other ethnic groups.

## Conclusions

To summarize, our analysis identified six risk proteins (NAMPT, TIE1, CBR1, PDE4D, PAFAH1B2, NCF2) and seven protective proteins (FSTL1, PTPN4, SOCS3, GZMB, GPC1, C1QC, FCGR3B) that are causally associated with MM risk. Additionally, we shed light on the role of these proteins in MM, prioritized the identified proteins, and evaluated their feasibility for drug development. These findings hold promise for advancing the integration of genome and proteome data to discover new drug targets for the treatment of MM. Further studies were warranted to explore the mechanisms through which the potentially causal proteins influence the risk of MM.

### Supplementary Information

**Additional file 1:** **Table S1. **Detailed information for data source of confounders used in the current study. **Table S2.** Genetic variants for Mendelian randomization analysis of the associations between causal risk proteins and multiple myeloma. **Table S3.** Mendelian randomization results of causal risk proteins on multiple myeloma. **Table S4. **Information of genetic variants for MR-BMA analysis. **Table S5. **Test of influential genetic variants in MR-BMA. **Table S6. **Test of outlying genetic variants in MR-BMA. **Table S7. **Top 10 models ranked by the model posterior probability after model diagnostics. **Table S8. **Meta-analysis for the effect of genetically predicted causal risk proteins on the risk of MM from FinnGen and UK Biobank. **Table S9. **Genetic variants for Mendelian randomisation analysis of the associations between multiple myeloma and significant proteins. **Table S10. **Mendelian randomization results of multiple myeloma on significant proteins. **Table S11. **Mendelian randomization of blood proteins on multiple myeloma using only variants in the cis-region of the gene encoding the blood proteins.

## Data Availability

GWAS data of plasma proteome are publicly available to researchers at http://www.phpc.cam.ac.uk/ceu/proteins/. GWAS data of MM of individuals from FinnGen was derived at https://www.finngen.fi/en/access_results and GWAS data of MM of individuals from UK Biobank was derived at https://pheweb.org/UKB-SAIGE/pheno/204.4. The sources for downloading GWAS data of confounders were presented in Additional file 1: Table. S1. Further information is available from the corresponding author upon request.

## Abbreviations

C1QC: Complement C1q subcomponent subunit C
CDK6: Cyclin-dependent kinase 6
CBR1: Carbonyl reductase 1
CI: Confidence interval
FCGR3B: Fc-gamma receptor III-B
FDR: False discovery rate
FSTL1: Follistatin-related protein 1
GPC1: Glypican 1
GIANT: Genetic investigation of anthropometric traits
GSCAN: GWAS and sequencing consortium of alcohol
GSMR: Generalized summary-data-based Mendelian randomization
GWASs: Genome-wide association studies
GZMB: Granzyme B
HEIDI: Heterogeneity in dependent instruments
ICD: International Classification of Diseases
IVs: Instrumental variables
IVW: Inverse–variance weighted
JAK-STAT: Janus kinase-signal transducer and activator of transcription
KEGG: Kyoto encyclopedia of genes and genome
LMM: Liner mixed model
LD: Linkage disequilibrium
MACE: Model averaged causal effects
MIP: Marginal inclusion probability
MM: Multiple myeloma
MR: Mendelian randomization
MR-BMA: Mendelian randomization-Bayesian model averaging
NAMPT: Nicotinamide phosphoribosyl transferase
NCF2: Neutrophil cytosol factor 2
OCs: Osteoclasts
OR: Odds ratio
PAFAH1B2: Platelet-activating factor acetylhydrolase IB subunit beta
PDE4D: CAMP-specific 3',5'-cyclic phosphodiesterase 4D
PTPN4: Protein tyrosine phosphatase non-receptor type 4
SERPINB9: Proteinase inhibitor 9
SNPs: Single nucleotide polymorphisms
SOCS3: Suppressor of cytokine signaling 3
TIE1: Tyrosine kinase with immunoglobulin-like and EGF-like domains 1
